# Two Cases of Cysts of the Iris, Etc.

**Published:** 1873-08

**Authors:** A. W. Calhoun

**Affiliations:** Vienna, Austria, Professor of Diseases of the Eye and Ear, Atlanta Medical College


### TWO CASES OF CYSTS OF THE IRIS, WITH OPERATIONS FOR THEIR REMOVAL.

BY A. W. CALHOUN, M.D., VIENNA, AUSTRIA,

*Professor of Diseases of the Eye and Ear, Atlanta Medical College.*

In February, 1873, Julius Klein, aged fifteen, came into the Clinic of Prof. Arlt for the relief of an affection of his right eye, and by his father the following short history in reference to his disease was communicated :

In October, 1871, while playing with his school-mates, he received a blow in his right eye from a staff. Comparatively little pain was felt immediately after the accident, but, in the course of a few days, the pain increasing and the inflammatory symptoms becoming more threatening, he was forced to confine himself to his room and bed for near eight weeks, at the end of which time the acute or direct effects of the injury had disappeared. Vision being, however, to a great extent lost, an oculist in Wuerzburg was consulted, just one year after the accident, when some operation was performed with good result, as the boy was thereby soon enabled to resume his studies without hindrance. As to the nature of the operation, no information could be given, nor could any examination of the eye, in its present state, contribute to any positive conclusion, on account of the changes that have taken place since that time. For several months past, vision has again been failing, and this circumstance, together with a renewal of the pain in the eye, and a continuous and troublesome photophobia and lacrimation, brings the patient to this clinic, with the eye at present in the following condition:

The lids are normal, but held closely together, the bulbus itself swimming in tears. Conjunctiva bulba is moderately injected around the cornea, slight ciliary injection being also present. In the upper part of the cornea, both to the inner and outer sides, are small cicatrices, and directly behind them a water-clear bladder with transparent walls and of the size of a large pea, springing from the peripheral edge of the iris, occupies the upper and inner part of the anterior chamber, reaching beyond the middle of the pupilla toward the outer edge of the chamber. The anterior wall of the cyst rests upon the cornea, or the descemetian membrane, producing a light, delicate opacity of the same. The posterior wall presses upon the iris, forcing it backward into a dish-shaped figure. The pupilla is recognized near the edge of the cyst, and partially covered by it, as a narrow, perpendicular crevice, closed by a thin, white mass. The iris tissue, or stroma, itself is much thinned, and somewhat atrophic, and along its superior periphery is a small orifice, caused by this portion of the iris becoming torn loose from the ciliary body (irido-dyalisis) and dropping down, as a direct consequence of the blow. The tension of the bulbus is not altered, but in the region of the ciliary bodies, the eye is sensitive, a symptom that these have begun to partake of the slight chronic inflammation existing in the anterior portion of the bulbus.

February 16th.—Cyst operated upon by puncturing the anterior chamber, through the upper and inner cornea-scleral junction, by means of an ordinary curved iridectomy knife, the blade passing through the anterior wall of the cyst, the posterior wall was seized and drawn out and cut away. This step of the operation was rendered quite easy and simple, since, as the aqueous humor flowed out, the cyst, now also emptied of its contents, was, in part, washed outward through the wound. The eye received the bandaging usual after such operations and the patient placed in a darkened room.

February 17th.—The corneal wound has healed, the anterior chamber regaining its normal depth, etc., and the narrow pupilla coming more clearly into view.

February 20th.—No appearance of the cyst, nor any signs of an inclination to return, as is the case mostly under such circumstances; the iris has retained its natural color, and the cornea has lost its cloudiness resulting from the contact with the cyst against its posterior surface.

February 24th.—To dilate the pupil, atropine has been used, and to-day it is much larger and of an oval form, and by aid of a concentrated light from the side, (focal light,) the membrane formerly filling the narrowed pupilla is seen to be cataracta secondaria, the remains of the cataractous lens, absence of which is now by widened pupil and focal light to be plainly discerned. It was, no doubt, for the removal, by extraction or discession, of the lens, it having become cataractous from the injury, that the operation in Wuerzburg was made. Under the influence of the atropine the pupil has been much widened, and through contraction of the muscular fibres of the iris, one or more openings have been torn in the cataracta secondaria, sufficiently large to allow the passage of rays of light into the interior of the eye, thus restoring him to as complete vision as the loss of the lens will allow, and also permitting an examination with the ophthalmoscope, which demonstrates the interior of the bulbus to be as normal on the one side as on the other. The removal of the cyst has, in all respects, been successful, and by the systematic examination of the vision several days following this, prior to patient's dismissal, it was found in right eye H., $\frac{1}{3\frac{1}{2}}$, (total hypermetropia,) vision, $\frac{20}{70}$; left eye, Hm., $\frac{1}{30}$, (manifest hypermetropia,) vision, 20/2[illegible]; whereas, upon entering the clinic the right eye had only a perception of light. To those conversant with the examination for anomalies of refraction, etc., these figures will be readily understood.

March 13th.—Patient presented himself to-day, before leaving for his home, with the eye remaining in the same favorable condition as when dismissed. No appearances of a return of the cyst.

Second Case.—Florian Eder, aged thirty-three. Fifteen years ago a small piece of iron flew with great force into his right eye, producing a free flow of blood and much subsequent inflammation, which ceased under appropriate treatment—the latter again returning at various intervals upon slight exposure, each time leaving the eye in a less useful condition. A few months ago, after one of these repeated inflammatory paroxysms, (chronic iritis, or rather irido-cychitis,) the vision was so far destroyed, and the eye so sensitive to the influence of light, that he was obliged to cease work, and to apply to the clinic on the 12th of February, 1873, for assistance.

His condition on entering is as follows: Lids of right eye somewhat swollen, and ciliary injection around the cornea, the two symptoms taken together denoting an internal inflammatory process. In the cornea and to its outer side is an old cicatrix, the result of the wound, and in the immediate neighborhood the cornea is very vascular. Directly underneath the cicatrix, in the interior chamber, is a clear, transparent bladder, also of the size of a large pea, fastened to the iris with an apparently small, thin base, and flattened between the opposing surfaces of the iris and cornea. A portion of the iris opposite the cicatrix has become united with the cornea, (anterior synechia,) and to a slight extent is the adjoining periphery torn from the ciliary bodies, as in the first case—iridodialysis. The iris is also slightly swollen, the pupilla being closed by a gray exudative membrane, the result of the iritis, and the aqueous humor moderately clouded. Perception of light is good, recognizing promptly the movements of the hand at a distance of thirty feet.

February 17th.—Punctured the anterior chamber, as in the first instance, in the lower corneo-scleral junction, the knife passing at the same time into the cavity of the cyst. The attempt to draw the walls of the cyst through the external wound was not so successful as before, since the walls were atrophic, and firmly adhered to the iris, and tore upon the slightest pulling, so that it was brought away only in small pieces.

February 19th.—No unpleasant symptoms dependent upon the operation, but the cyst has again filled to about half its original size.

February 24th.—Without further interference the cyst has of its own accord bursted, leaving only a slight trace of its existence. A light iritis has for the last few days given the patient some pain, but succumbed under treatment of leeches and ice-poultices. This, however, has no connection with the operation for the removal of the cyst, but is more a fresh outburst of the long existing chronic inflammation of the iris, which, with its consequent occlusion of the pupilla, was the real cause of the patient's application for relief, he being not the least influenced by the presence of such an abnormal formation as an iris cyst, which he observed shortly after his injury, but which having reached in a short time its present size and shape, and so remaining, had never troubled him in the least degree, and hence he had never sought advice in reference to it, and was much surprised when informed that an operation must first be made for the removal of this, before his chief complaint could be attacked—viz, the loss of sharp or acute vision.

March 10th.—The acute iritis having disappeared, and the remnant of the walls of the cyst having shrunken to a small, unimportant mass, an iridectomy is now made, to substitute an artificial pupil for the natural one, which is occluded by an exudative mass, and also to guard against further internal processes, apt to ensue from extensive posterior synechia, which are present in this case.

March 22d.—Since the iridectomy the patient has had several slight, transitory attacks of his chronic affection, the irido-cyclitis, but under appropriate treatment they were easily overcome. The iridectomy has been of but little service, as the artificial pupil has also closed through an exudative membrane, the product of the light inflammatory attacks, and as any flattering success from an immediate repetition of the operation is doubtful, it is thought best to postpone any further interference, especially since the most interesting part of his disease, the cystic formation, has been completely set aside, and, as yet shows no tendency to return. From these previous and recent repeated attacks, the bulbus has become much softer than the normal, the usual result of irido-cyclitis, it often going to such an extent as to end in pthisis-bulbi.

May 1st.—Iridectomy is again made for the substitution of an artificial pupil, and, so far, with success. Still no indication of a reappearance of the cyst.

The very favorable result of the operative interference in these two cases of iris cysts, and their comparative rarity, have induced me to report them, with the view of affording some new ideas as to their treatment and removal, to any who may have such in their practice. That amongst twelve or fifteen thousand eye patients treated in this hospital during the last two years only three cases of cysts have appeared, is an item clearly demonstrating that it is an affection but seldom met with, and that such a fact is not merely a coincidence, is further supported when we remember how rarely we read reports bearing upon the disease, and the remedies for its cure. The two cases just described are another evidence that the large majority, if not all cysts of the iris, are of traumatic origin, occurring often directly after the injury, again at more or less extended intervals. Mostly we can trace their beginning to some blow upon the exterior of the bulbus, the iris being only indirectly injured, as was the case in one of the instances just reported, where, from the force of the blow, the periphery of the iris was partially ruptured in is connection with the ciliary bodies. Such cystic growths have also bean known to follow certain operations for the removal of a cataractous lens, as by discission and the usual flap operation, where the iris had been very probably pressed upon and bruised, soon declaring its susceptibility to inflammation, through the least mishandling, by the appearance upon some portion of its surface of a small vesicle, sometimes perfectly transparent, again more or less opaque, having, in most cases, a small narrow pedicle as the connecting link to the substance of the iris.

As before stated, the presence of such formations calls forth but little disturbance in the majority of instances, unless by their increase they extend into the area of the pupil and cut off the entrance of light into the interior of the eye. Occasionally, however, a very moderate amount of iritis is induced by their constant contact with and pressure, in full developed cases, upon the surface of the iris, and one or two instances have been known where sympathetic inflammation of the sound eye was brought about by their presence, which, happily, in each case, rapidly vanished as soon as the cyst had been extirpated.

As relates to treatment, the management and cure of the above two cases speak for themselves, and portray unmistakably the simplest and surest manner of dealing with such formations. In nearly all the previous cases, the iridectomy of that portion of the iris from which the cyst sprang has at the same time been made, thus removing at once the cyst with its iris attachment, supposing this method to make assurance doubly sure, in so far as preventing a return of the cystic growths, but, as under such circumstances an iridectomy has its disadvantages, it is well to avoid it where possible. Guided by such views, these cases were operated upon as above described, viz: by complete removal of the cyst together with its base or pedicle. or by breaking down its walls, and bringing them away piece by piece, leaving the iris, as a whole, intact, and so far the results have been absolutely good. Under any and all treatment, a return here and there takes place, also can an iritis follow, but neither of which can in this or that instance be attributed to mismanagement or a wrong mode of operating.

It must be borne in mind that the iridectomy in one of the above cases was not made to destroy the cyst, but after the cyst had disappeared, for the setting aside of the occlusion of the pupilla, a consequence of a former irido-cyclitis.

